# PUTTING NEGOTIAGE IN ACTION: APPLYING SKILLS FROM AN AI-BASED NEGOTIATION TRAINING FOR CAREGIVERS OF OLDER ADULTS

**DOI:** 10.1093/geroni/igae098.3137

**Published:** 2024-12-31

**Authors:** Celie Joblin, Alaine Murawski, Marianne Tschoe, Vanessa Ramirez-Zohfeld, Johnathan Mell, Jeanne Brett, Lee Lindquist

**Affiliations:** Northwestern University, Chicago, Illinois, United States; Northwestern University, Evanston, Illinois, United States; Northwestern University, Evanston, Illinois, United States; Northwestern University, Evanston, Illinois, United States; Custom Technologies LLC, Orlando, Florida, United States; Northwestern University, Evanston, Illinois, United States; Northwestern University, Evanston, Illinois, United States

## Abstract

Family caregivers provide crucial support for older adults, yet this can sometimes lead to conflict. We created NegotiAge, an online AI-based negotiation training to help caregivers resolve conflicts. Through instructional resources and practice with AI-based avatars, caregivers learn to navigate conflicts. We characterized how family caregivers utilized NegotiAge in addressing real-life conflicts with their older adults. Family caregivers participated in a randomized controlled trial of the NegotiAge intervention. A month later, they were surveyed with open-ended questions about their use of knowledge and skills gained with NegotiAge with the older adult they support. Two coders analyzed the data using constant comparative thematic analysis. 119 caregivers (mean age 53.5 yrs, 75.6% female, 32.8% Black) completed the training and the one-month survey. 73% (n=119) of participants managed conflicts related to caregiving in the month following NegotiAge. Conflict themes involved: 1) health-related concerns, 2) daily living tasks, 3) finances, 4) acceptance of assistance, and 5) familial duties. 86% (n=119) of participants reported handling conflicts differently because of the NegotiAge training. Themes that surfaced included: 1) changing behaviors away from power dynamics to shared interests, 2) uncovering the causes of conflict, and 3) enhancing communication. Participants utilized their negotiation skills in various contexts, including professional environments, retail, real estate, and other relationships. NegotiAge positively influenced how family caregivers manage conflicts. They applied skills acquired from NegotiAge to manage conflicts with older adults, family, and others. Subsequent research will explore NegotiAge’s impact on caregiver burden, stress, and the care of older adults.

